# Metataxonomic insights into lactic acid bacteria diversity in artisanal coalho cheese from the Caatinga biome

**DOI:** 10.1371/journal.pone.0352903

**Published:** 2026-07-09

**Authors:** Ruana Célia Gomes De Morais Costa Rodrigues, Emmanuella de Oliveira Moura Araújo, Samira Teixeira da Silva, Gustavo Felipe Correia Sales, Danielle Cavalcanti Sales, Mauricio Tolstoi do Santos Ferreira, Cláudio Vaz Di Mambro Ribeiro, Luis Medeiros de Lucena, Claudio Cipolat-Gotet, Maria Taciana Holanda Cavalcanti, Adriano Henrique do Nascimento Rangel

**Affiliations:** 1 Postgraduate Program in in Animal Production, Federal University of Rio Grande do Norte, Macaíba, Brazil; 2 Academic Unit Specialized in Agricultural Sciences, Federal University of Rio Grande do Norte, Macaíba, Brazil; 3 Department of Animal Science, Federal University of Paraíba, Areia, Brazil; 4 Postgraduate Program in Climate Sciences, Federal University of Rio Grande do Norte, Macaíba, Brazil; 5 Veterinary and Animal Science School, Federal University of Bahia, Salvador, BA, Brazil; 6 Department of Veterinary Science, University of Parma, Parma, Italy; 7 Department of Morphology and Animal Physiology, Federal Rural University of Pernambuco, Recife, Brazil; Universidad Autonoma de Chihuahua, MEXICO

## Abstract

Artisanal raw-milk cheeses are complex microbial ecosystems that reflect local environments through spontaneous fermentation driven by lactic acid bacteria (LAB), processing practices, and the quality of raw materials. The Caatinga biome, a unique semi-arid ecosystem in Northeastern Brazil, presents distinctive environmental and cultural conditions which can significantly influence the microbial composition of traditional dairy products. This study aimed This study aimed to characterize the bacterial community, with emphasis on lactic acid bacteria (LAB), in artisanal Coalho cheese produced in the Seridó region using 16S rRNA gene-based metataxonomic analysis. A total of 32 cheese samples from eight municipalities were collected and homogenized into a representative composite sample. DNA was extracted and the V3–V4 region of the 16S rRNA gene was sequenced using the Illumina MiSeq platform (paired-end 2 × 300 bp). The microbial community was dominated by LAB, particularly Enterococcus (~25%), followed by Lactococcus, Streptococcus, and Leuconostoc. The predominance of these genera reflects the spontaneous fermentation process typical of artisanal raw-milk cheeses and highlights their role in acidification, flavor development, and microbial stability. The microbial community was dominated by LAB, particularly *Enterococcus* (~25%), followed by *Lactococcus*, *Streptococcus*, and *Leuconostoc*. The predominance of these genera reflects the spontaneous fermentation process typical of artisanal raw-milk cheeses and highlights their role in acidification, flavor development, and microbial stability. This study provides the first metataxonomic insight into the microbiota of artisanal Coalho cheese produced in the Seridó region of the state of Rio Grande do Norte, highlighting its microbial richness and biotechnological potential, and supporting its valorisation and the sustainable development of regional cheese production. Although the use of pooled samples limits the assessment of intra-regional variability, the results establish a foundational understanding of the microbial community structure and support future investigations focused on functional characterization, food safety, and process standardization.

## Introduction

Artisanal coalho cheese, a hallmark product of the Seridó region in Rio Grande do Norte, Brazil, holds significant economic, cultural and gastronomic relevance. Its production relies on traditional techniques passed down through generations, primarily involving raw bovine milk and rennet for coagulation, followed by cutting, draining, molding and salting [1]. The cheese’s firm yet flexible texture, coupled with a subtly salty flavor, imparts unique sensory characteristics widely recognized regionally and appreciated across Brazil [2].

Unlike other artisanal cheeses from Seridó, such as butter cheese, coalho cheese coagulation is rennet-dependent rather than solely milk acidification. However, manufacturing practices vary considerably among producers, reflecting differences in raw materials, milk handling, and processing techniques, which often leads to inconsistencies in product quality and standardization [3,4].

Despite its traditional value, comprehensive scientific studies detailing the microbiological and technological composition of artisanal coalho cheese remain scarce, thereby limiting standardization and quality control efforts [3]. Previous research indicates significant variability in protein, fat, and moisture content among artisanal cheeses from the Northeast, underscoring the critical need for studies to characterize and standardize manufacturing technology without compromising the traditional properties of coalho cheese [2]. In recent years, there has been increasing interest from state governments, rural producers, health inspection agencies, and research institutions in regulating and promoting artisanal cheese production in the Seridó Potiguar region. Molecular analyses and detailed production methods are essential for accurate diagnoses of the production system, supporting public policies, fostering certification programs, and strengthening the region’s territorial identity [3,5].

Addressing this imperative, the present study aims to contribute to the valorization and sustainable development of artisanal coalho cheese. Our objective is to conduct a comprehensive metataxonomic characterization of the lactic acid bacteria (LAB) diversity present in artisanal coalho cheese from the Seridó region, utilizing 16S rRNA gene sequencing. By establishing a descriptive baseline of the regional microbial signature, we intend to provide crucial information for safety assessment, quality enhancement, and the preservation of the traditional characteristics that confer its unique regional identity.

## Materials and methods

### Characterization of the study site

The study was conducted in the Seridó region of the state of Rio Grande do Norte, located within the Caatinga biome. This region is characterised by a semi-arid climate, low rainfall and vegetation adapted to water scarcity. Seridó is a prominent part of the dairy basin of Rio Grande do Norte (Brazil), recognized for its strong tradition of family farming and the production of artisanal cheeses, such as coalho and butter cheeses, which constitute the basis of the local sociocultural and economic identity [6]. The selection of this study area is justified by the regional relevance of dairy farming and cheese production as strategies for adaptation to semi-arid conditions, as well as by their role in strengthening the regional economy and preserving traditional knowledge associated with the state’s food culture.

### Collection and preparation of artisanal coalho cheese samples

**Fig 1:**
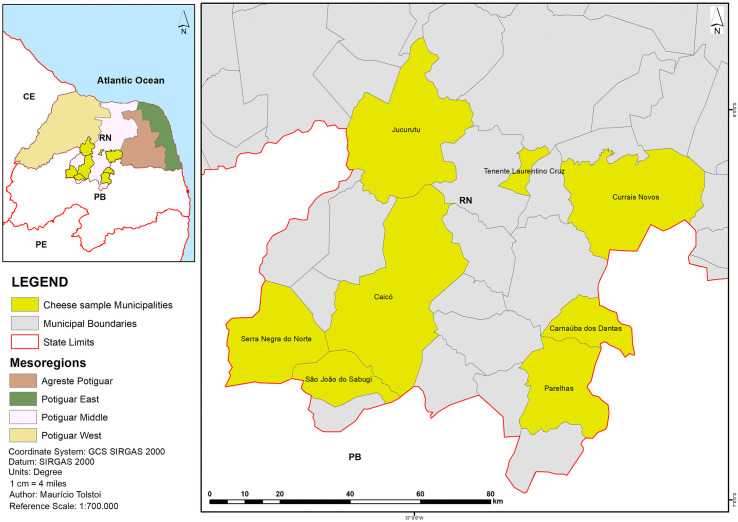
Geographical location of artisanal Coalho cheese producing municipalities in the Seridó region, Rio Grande do Norte, Brazil. The map highlights the eight municipalities (Caicó, Currais Novos, Parelhas, São João do Sabugi, Serra Negra do Norte, Ipueira, São José do Seridó, and Jardim do Seridó) where the 32 cheese samples were randomly collected between February and June 2024. The cartographic mesh is based on open-access data from IBGE and IDEMA, processed in QGIS software to represent the origin of the microbial “terroir” studied.

Artisanal coalho cheese samples were purchased at random from local markets and production units across eight municipalities in the Seridó region of Rio Grande do Norte (Caicó, Currais Novos, Parelhas, São João do Sabugi, Serra Negra do Norte, Ipueira, São José do Seridó, and Jardim do Seridó) (Fig 1). The samples were collected between February and June 2024. Only cheeses clearly identified as artisanal products were included. To ensure representativeness while maintaining an exploratory and descriptive focus, samples were obtained in duplicate from different batches, totaling 32 units (pieces of approximately 500 g each, vacuum-packed). The collection of samples duplicated from different batches aimed to ensure representativeness of the regional microbial ‘terroir,’ accounting for variations in production dates and batch differences inherent to artisanal systems, rather than for comparative analysis between individual batches.

Samples were transported under refrigeration (4–7 °C) to the Milk Quality Laboratory (LABOLEITE/UFRN) for preparation. Upon arrival, the external surfaces of the packages were disinfected with 70% alcohol. The packages were opened aseptically using sterile instruments. Each sample unit was individually macerated and homogenized. For the metataxonomic analysis, 100 g aliquots from each of the 32 samples were combined into a single composite pool to provide a comprehensive regional microbial overview. This pooling strategy was designed for a descriptive characterization of the regional “terroir” rather than for comparative ecological inferences between specific groups or municipalities. The pooled samples were stored at −20 °C and subsequently transported to Neoprospecta Microbiome Technologies (Florianópolis/SC) under biosafety protocols for molecular analysis. Physicochemical parameters were evaluated concurrently on the individual samples (n = 32).

### Physical-chemical composition of cheese samples

The physicochemical analyses were performed in triplicate following official methodologies. Moisture, pH, and total titratable acidity were determined according to the procedures described by the Official Methods for the Analysis of Animal Products – MAPA, 1st edition (2022) [7] and Adolfo Lutz Institute (IAL) [8]. Fat, protein, and ash contents were determined according to AOAC methods [9].

### Determination of pH, and titratable acidity

A calibrated digital pH meter (Lucadema, São José do Rio Preto-SP) was used for measurements, with the electrode inserted directly into the homogenized samples (20g). Calibration was performed using pH 4.0 and 7.0 buffer solutions. Titratable acidity was determined by titration of 10 g of sample with 0.1 N NaOH, using phenolphthalein as an indicator, and results were expressed as a percentage (%) of lactic acid [9].

### Chemical composition and energy value

Moisture content was determined by drying 3 g of sample in a MOC63 moisture analyzer (Shimadzu) at 160 °C until constant weight. Fat content was determined using the Gerber method, and ash content was measured by incineration in a muffle furnace at 550 °C. Total nitrogen was determined by the Kjeldahl method, and crude protein was calculated using a conversion factor of 6.38 [9]. The protein percentage was calculated by the equation:

In which:


$$\begin{array}{c} P\;(\%)=\;\frac{K\;x\;V\;x\;Fator}{P} \end{array}$$

K = Fc × 0.0014 × 100 (Fc: correction factor for hydrochloric acid solution).

V = volume of hydrochloric acid used in titration (mL).

Factor = conversion of nitrogen to protein.

*P* = sample mass (g).

Carbohydrates were estimated by difference. To address analytical closure errors where the sum of components exceeded 100%, a proportional solids normalization was applied: measured solid components (protein, fat, and ash) were adjusted so their sum matched the total solids content (100 − moisture), preserving their relative proportions. Energy value (kcal/100 g) was estimated using Atwater factors (4 kcal/g for protein and carbohydrates, 9 kcal/g for fat). Statistical analysis of physicochemical data was performed using descriptive statistics (mean, SD, and ranges) to characterize the 32 individual samples.

### DNA extraction and metataxonomic characterization

The bacterial composition was characterized by sequencing the V3–V4 hypervariable region of the 16S rRNA gene. DNA was extracted from 25 g replicates of the pooled sample using a magnetic bead-based method according to the Neoprospecta Microbiome Technologies internal protocol. This region was selected for its high taxonomic resolution and classification accuracy in dairy matrices, allowing for the identification of key genera such as *Lactococcus*, *Streptococcus*, and *Enterococcus*.

### Library preparation and sequencing

**Fig 2:**
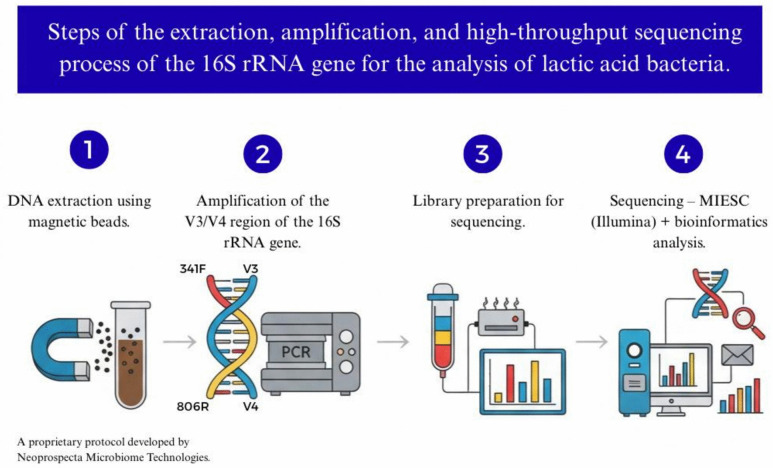
Methodological workflow of metataxonomic analysis of Coalho cheese microbiota. The scheme details the steps from genomic DNA extraction extraction, PCR amplification of the V3-V4 region, paired-end sequencing (2x300 bp), and bioinformatics processing on the QIIME2 platform.

Amplification of the V3–V4 region was performed via PCR using the primers 341F and 806R [5]. Negative controls (NEC) were included in all library preparation steps (PCR1 and PCR2). Sequencing was performed on the Illumina MiSeq platform using a paired-end 2 × 300 bp format with the iSeq 100 i1 V2 300-Cycle kit (Illumina Inc., San Diego, CA, USA). The methodological workflow is illustrated in Fig 2 (Table 1).

**Table 1:** *Primer* sequences used for amplification of the V3–V4 region of the 16S rRNA gene*.*

PRIMER	SEQUENCE 5′–3′
Bakt_341 FW	CCTACGGGNGGWGCWGCAG
Bakt_806 RV	GGACTACHVGGGTWTCTAAT

### Bioinformatics and taxonomic assignment

Raw sequences in FASTQ format were processed using the QIIME2 platform. Reads underwent pair joining, size filtering (>240 bp), quality assessment (PHRED > 30), and dereplication with the VSEARCH tool. Amplicon sequence variants (ASVs) were determined by clustering with 99% similarity. Taxonomic assignment was performed using the curated NeoRef database (Neoprospecta Microbiome Technologies, Brazil), validated by technical experts. This approach ensured accurate identification of the lactic acid bacteria and opportunistic taxa.

Raw 16S rRNA sequencing data have been deposited in the NCBI Sequence Read Archive (SRA) under BioProject accession PRJNA1426985. Given the exploratory and descriptive nature of this study, which aimed to characterize the regional microbial “terroir” through a pooled sampling approach. All samples were homogenized into a single composite pool prior to DNA extraction. This approach was adopted to provide a representative overview of the regional microbiota.

## Results and discussion

### Physicochemical properties of artisanal coalho cheese samples from the Seridó region in the state of Rio Grande do Norte

A total of 32 analytical determinations were used to characterize the physicochemical composition of artisanal coalho cheeses, revealing the average values described in Table 2. Moisture averaged 43.56% (±2.36), fat 25.03% (±3.38) and protein 30.77% (±3.59). Ash content was 4.58% (±0.53). After proportional normalization of solids, carbohydrates averaged 3.50% (±1.29) and the estimated energy value was 332 kcal/100 g. The variability observed among replicates reflects the inherent heterogeneity of artisanal production systems.

**Table 2:** Composition of artisanal curd cheeses (n = 32). Values expressed on a wet basis as mean ± SD (min.-max.).

Parameter	Mean ± SD	Min – Max
Moisture (%)	43.56 ± 2.36	38.92–48.27
pH	6.32 ± 0.40	5.52–6.87
Fat (%)	25.03 ± 3.38	18.50–29.50
Protein (%)	30.77 ± 3.59	26.02–37.75
Ash (%)	4.58 ± 0.53	3.56–5.58
Carbohydrates (%)*	3.50 ± 1.29	1.10–5.40
Energy (kcal/100 g)	332 ± 18	295–361

*Carbohydrates estimated by difference after proportional normalization of solids; Min.:Minimum; Max.:Maximum.

The proximate composition of the artisanal coalho cheeses analyzed in this study, with a mean moisture content of 43.56%, positions the product within the semi-hard cheese category. This finding is consistent with previous reports for coalho and other Brazilian artisanal cheeses, which typically exhibit moisture levels between 40% and 55% [10,11]. The observed homogeneity in moisture content, despite the artisanal production context, suggests a degree of inherent standardization in the manufacturing process.

The observed pH values ranging from 5.52 to 6.87 with a mean of 6.32, are within the range (5.5 to 6.5) typically reported for fresh curd cheeses [12]. These values suggest incomplete acidification, potentially attributable to the limited use of starter cultures or the short maturation period characteristic of these artisanal products [13].

Such pH condition can significantly influence the resident microbiota, cheese texture, and overall microbiological stability, thereby emphasizing the importance of identifying the natural lactic acid bacteria driving fermentation in these systems. This broad dispersion in pH values reflects the inherent heterogeneity of artisanal production, as previously documented [14], and may be linked to variations in initial milk fat content, draining procedures, and coagulation handling. This variability underscores how a lack of standardization in raw materials and processing directly affects the centesimal composition of the final product.

The mean fat content of 25.03% and protein content of 30.77% indicate a high-solids matrix with substantial nutritional density, characteristic of cheeses produced under traditional, small-scale conditions. These fat values align with the classification of medium-fat cheeses according to the Technical Regulation on Identity and Quality [15]. Similar fat contents, ranging from 25% to 29%, have been reported in artisanal coalho cheeses from the Northeast region such as those described [16], with variability often attributed to raw milk composition and differences in draining and pressing processes. The observed variation in lipid content in our samples [17] is consistent with the use of unstandardized raw milk, potential partial skimming, and diverse pressing methods. Notably, the average protein content in this study (30.77%) was higher than that typically found in industrially produced coalho cheeses, which range from 18% to 25% [18].

The ash content (4.58% on a wet basis) fell within the expected range for raw-milk cheeses, supporting the mineral contribution characteristic of products from semi-arid regions, where animal diet and water availability can affect milk composition. The observed lower carbohydrate content (3.5%) in this study, compared to the 5–7% commonly reported for coalho cheeses from other Northeast micro-regions, is consistent with near-complete lactose conversion during early fermentation. This lower value may also be attributed to the higher solids and protein levels in our samples, which proportionally reduce the residual carbohydrate fraction [19]. Additionally, spontaneous fermentation by native lactic acid bacteria in raw-milk cheeses can lead to extensive lactose utilization during early storage, resulting in reduced residual carbohydrate levels. Methodological differences, particularly the application of proportional solids normalization in this study to correct for analytical closure errors, may also contribute to the observed discrepancy, providing a more conservative and internally consistent estimation of carbohydrate content.

The estimated energy value (332 kcal/100 g) is consistent with data reported for semi-hard cheeses in Brazil and globally [20,21]. The variability observed between samples reflects the heterogeneity in raw materials, artisanal practices, and environmental conditions, typical of the production system based on the Caatinga. This variability highlights the importance of establishing compositional bases to support quality control, traceability, and future regulatory frameworks for artisanal cheeses in Brazil [14]. Overall, the composition indicates a nutritionally dense product with considerable variability among artisanal production units. This physicochemical profile helps characterize coalho cheeses from the Caatinga biome and complements the microbiological findings of this study. The heterogeneity observed among samples reflects the regional identity of artisanal production but may also influence product quality and stability. These results highlight the importance of adopting good manufacturing practices that preserve the cheese’s traditional attributes while supporting greater process standardization, quality consistency, and future technological improvements.

### Taxonomic structure and “Microbial Terroir” of Seridó Coalho cheese

**Fig 3:**
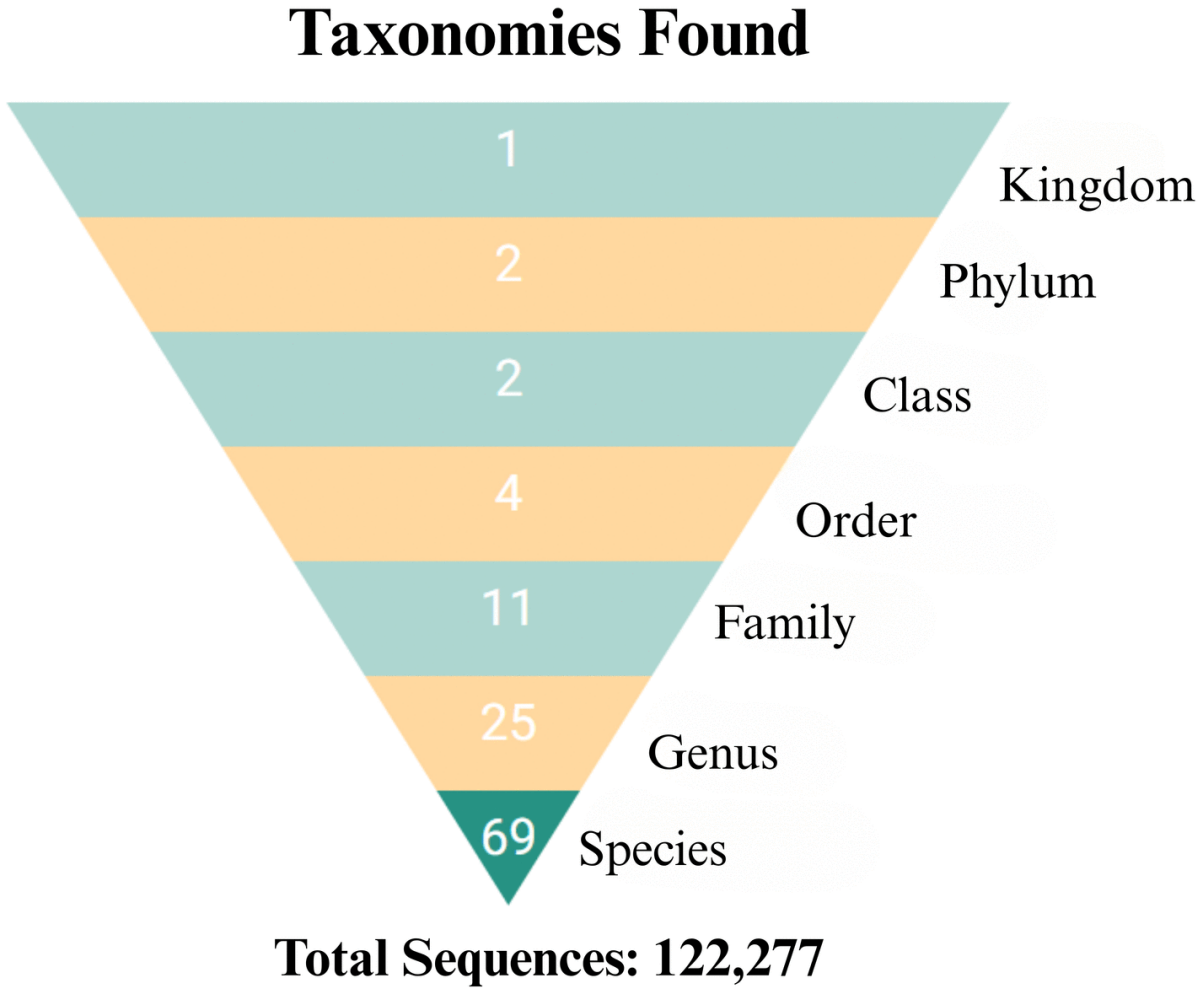
Hierarchical taxonomic distribution of identified sequences in artisanal Coalho cheese. The graph represents microbial diversity organized by Kingdom, Phylum, Class, Order, Family, and Genus. The analysis reveals the predominance of Firmicutes and Proteobacteria phyla, reflecting the complexity of the spontaneous fermentation ecosystem in raw milk under the climatic conditions of the Caatinga biome.

The metataxonomic analysis of samples of artisanal Seridó of Rio Grande do Norte Coalho cheese revealed a rich and taxonomically diverse microbiota composed of 69 bacterial species distributed across 25 genera, 11 families, 4 orders, 2 classes, and 2 phyla, all belonging to the Bacteria Kingdom (Fig 3).

Among the phyla identified, Firmicutes and Proteobacteria were the predominant groups, consistent with microbiological profiles reported in other fermented dairy products made from raw milk [22,23]. This predominance underscores the central role of the indigenous microbiota in fermentation, sensory development, and food stability, aligning with patterns observed in artisanal cheeses from other regions of Northeast Brazil and Europe [24]. Using culture and genetic sequencing techniques, [25] showed that the microbiota of the cow’s udder skin shares phyla also detected in this study, such as Firmicutes and Proteobacteria. These findings suggest that the udder skin may act as an important source of microorganisms for raw milk, directly influencing the microbial composition of the product used to make artisanal cheeses. Technologically relevant genera such as *Lactococcus*, *Lactobacillus*, and *Enterococcus* stand out in the Firmicutes phylum; *Lactococcus* spp. plays an essential role in the initial acidification of milk, *Lactobacillus* spp. contributes to maturing and developing sensory characteristics, and *Enterococcus* spp. participates in forming the aromatic profile and stability of the cheese, reinforcing the importance of lactic microbiota in the identity and technological quality of artisanal Coalho cheese.

These genera perform additional functions, including producing bacteriocins and competitively excluding opportunistic microorganisms, modulating the microbial composition of the final product [26]. These lactic acid bacteria also participate in the synthesis of organic acids, aromatic amino acids, and exopolysaccharides, directly influencing texture, flavour, food safety, and conferring a potential probiotic effect. Although Proteobacteria in the phylum include microorganisms of limited technological interest, some species have health implications, often associated with environmental contamination, hygiene failures, or manufacturing defects [25].

A critical aspect of our study design was the intentional use of pooled samples from eight different municipalities (Fig 1). While this approach does not allow for the assessment of intra- producer or intra-regional variability, it was specifically designed to capture a representative “macro” signature of the Seridó region. By collecting samples from multiple sites, we aimed to define the overarching “microbial terroir” of the Caatinga biome a unique microbial signature resulting from the interaction between traditional practices, the semiarid environment, and regional raw materials [26–28]. This broad regional representation establishes a comprehensive baseline that strengthens the identification of core taxa defining the artisanal queijo de coalho of Rio Grande do Norte. Future studies with higher spatial resolution and longitudinal sampling will be necessary to further dissect the drivers of microbial shifts across individual production units.

The detailed knowledge of the microbiota through metataxonomic approaches offers opportunities for technological innovation, including optimising artisanal processes, developing indigenous starter cultures, and strengthening food safety, without compromising the sensory characteristics which make Seridó coalho cheese a unique and valued product.

### Quantitative interpretation of Lactic Acid Bacteria (LAB) diversity

**Fig 4:**
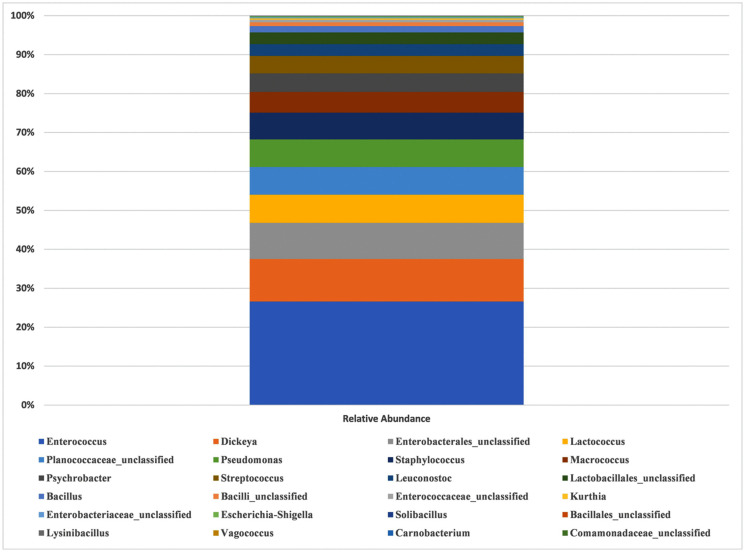
Relative abundance (%) of bacterial genera detected in artisanal Coalho cheese from Seridó. The bar graph illustrates the composition of the microbial community, highlighting the dominance of *Enterococcus* (approx. 25%), followed by *Lactococcus, Streptococcus,* and *Leuconostoc*. Taxa were identified based on amplicon sequence variants (ASVs) with 99% similarity. Genera with abundance less than 1% were grouped into the “Others” category to facilitate visualization of the main microbiota structure.

The microbial community identified (Fig 4) was highly diverse and predominantly composed of lactic acid bacteria (LAB), a group functionally associated with the fermentation of raw milk and the maintenance of product microbiological safety [29]. This diversity is characteristic of spontaneous fermentation systems, directly influenced by factors such as the absence of pasteurization, the heterogeneity of practices among producers, and the recurrent use of reused utensils and surfaces, which favor the formation of biofilms and the establishment of an autochthonous microbiome adapted to the artisanal production environment [30]. Unlike previous descriptive reports, our quantitative data (Fig 4) highlights specific taxonomic drivers of the Seridó cheese profile.

*Enterococcus* was the dominant genus, representing approximately 25% of the total bacterial community. This quantitative dominance underscores its robust adaptation to the semiarid processing conditions, characterized by pH fluctuations (5.5–6.8) and salt concentrations. This high prevalence is consistent with previous studies on artisanal raw-milk cheeses [31]. While *Lactococcus* (the second most abundant genus) drives initial milk acidification due to rapid acidification capabilities [32], the sustained high abundance of *Enterococcus* particularly *E. durans* and *E. faecium* suggests a significant role in proteolysis and lipolysis during early maturation, shaping the unique regional flavor [33]. *Enterococcus* also contributes to acidifying milk, coagulating casein, and inhibiting undesirable microorganisms [21], with many strains producing bacteriocins effective against food pathogens like *Listeria monocytogenes*, *Salmonella* spp., and *Staphylococcus aureus* [34]. The probiotic potential of certain *Enterococcus* strains from artisanal products, such as *E. faecium* and E. *durans,* has also been noted [33]. The dominant presence of Enterococcus in this study aligns with patterns observed in other Brazilian artisanal cheeses (e.g., Canastra, Marajó), suggesting its role as an ecological marker of Brazilian artisanal cheeses [35]. However, it is important to note that *Enterococcus* also includes opportunistic species with a history of nosocomial infection, such as multidrug-resistant *E. faecalis* and *E. faecium* [36,37]. *Lactococcus* spp., represented a substantial portion of the microbial community, approximately 20–25% (Fig 4), identified as the second most abundant genus, plays a crucial role in the initial stages of fermentation. Their rapid acidification capabilities are essential for controlling undesirable microorganisms and contributing to characteristic texture and flavor development [38]. Species like *Lactococcus lactis* produce volatile and aromatic compounds (diacetyl, acetaldehyde, acetone), which give cheese distinctive sensory characteristics [39]. From a technological perspective, *L. lactis* also produces bacteriocins (e.g., nisin) with antimicrobial activity against pathogens, contributing to cheese safety and stability [40]. This multifunctionality makes *Lactococcus* a strategic ally in artisanal cheese production. *Leuconostoc* spp. were also detected in significant proportions, contributing to aroma and flavor complexity through diacetyl production and texture modulation via CO₂ formation [39,41]. *Leuconostoc mesenteroides* is particularly valued for generating CO₂ responsible for forming eyes and producing aromatic metabolites, such as diacetil, which contribute to the characteristic flavor of artisanal cheeses [28]. Its presence is recurrent in naturally fermented cheeses and is considered a marker of regional microbial identity [29]. Studies on artisanal cheeses from Serra da Canastra and Minas Artesanal also identified *Leuconostoc* as a main genus, associating its action with aroma formation and sensory complexity [42,43].

The *Streptococcus* spp., observed at an approximate relative abundance of 10–15% (Fig 4), represents another significant component of the microbiota in artisanal coalho cheese samples from the Seridó region of Rio Grande do Norte. While *Streptococcus thermophilus* is known for its application in commercial starter cultures [44], its presence in artisanal raw- milk cheeses like those from Seridó, Serro (MG), and Canastra (MG) may reflect an environmental origin or the milk itself, as the genus includes commensal or opportunistic species from ruminant mammary and oral tracts [45,46]. The identification of *Streptococcus* in artisanal Coalho cheese reinforces the influence of traditional practices and the absence of starter culture in shaping a spontaneous and regionalized microbial profile [47].

### Food safety implications and opportunistic taxa

The detection of genera such as Enterobacteriaceae, Escherichia-Shigella, Bacillus, Staphylococcus, and Dickeya (Fig 4) requires a critical evaluation of the food safety implications in artisanal Coalho cheese. These microorganisms are often associated with environmental contamination, hygiene failures during processing, or the raw material itself. The genus *Dickeya,* a plant pathogen, was unexpectedly abundant (11.2%). While not typically associated with dairy products, its presence may indicate environmental contamination from soil, water, or plant matter during milk handling or cheese production [48–50]. Although its direct impact on human health is not well-established in this context, its high relative abundance warrants further investigation as a potential indicator of broader environmental contamination.

It is crucial to acknowledge the limitations of 16S rRNA gene sequencing for assessing food safety risks. This DNA-based method does not differentiate between viable and non-viable cells, meaning the detection of a pathogen’s genetic material does not confirm the presence of active, infectious agents [34]. Furthermore, 16S rRNA sequencing often has limited resolution at the species or strain level, which is critical for distinguishing pathogenic from non-pathogenic variants (e.g., E. coli strains) [29]. Therefore, these findings should be interpreted as a first-step screening that highlights potential risks, rather than a definitive confirmation of a food safety hazard. Future studies should employ culture-based methods, whole-genome sequencing, and functional assays to assess the viability, pathogenicity, and toxigenic potential of these microorganisms.

A limitation of the current study is the use of pooled samples from different municipalities, which may mask intra-producer and intra-regional variability. However, this clustering approach was intentionally chosen to capture a representative “macro” signature of the Seridó region, establishing a foundational baseline for the “microbial terroir” of artisanal coalho cheese. Future studies with higher spatial resolution and longitudinal sampling will be necessary to further dissect the drivers of microbial shifts across individual production units.

**Fig 5:**
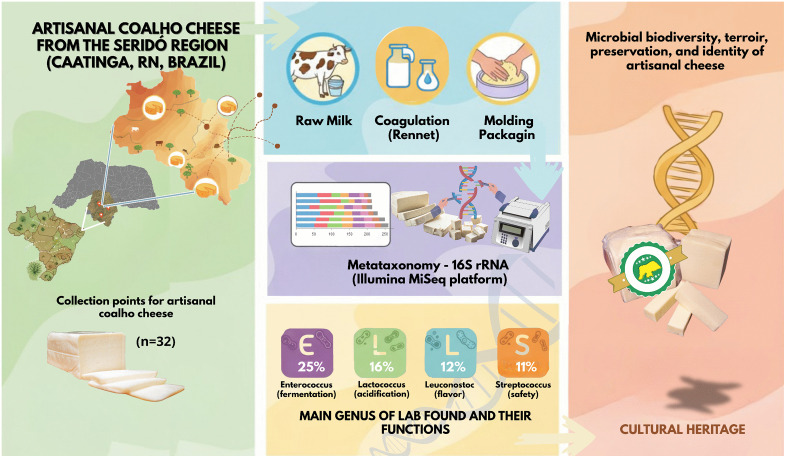
Illustration of the study, variables and main results found in the identification of lactic acid bacteria in artisanal Coalho cheeses from Seridó, RN (Caatinga biome).

This work provides the first in-depth molecular characterization of the microbial terroir of artisanal Coalho cheese from the Caatinga biome, offering essential baseline data for the valorization of this traditional product. By identifying the dominant lactic acid bacteria and critically evaluating the implications of opportunistic taxa, our findings contribute directly to the field of milk quality and food safety, supporting the development of Good Manufacturing Practices tailored to the unique conditions of the Brazilian semi-arid region. We believe this study represents a significant contribution to the understanding of traditional dairy systems and their sustainable development. The main results of the study are illustrated in Fig 5.

## Conclusion

This study provides a comprehensive overview of the microbial diversity and composition of artisanal coalho cheese from the Caatinga biome. The results highlight the importance of the local environment and production practices in shaping the microbial terroir of this traditional cheese. The findings of this study can be used to support the valorisation and sustainable development of regional cheese production, as well as to develop strategies for improving the quality and safety of artisanal coalho cheese.
